# Caring to deny, confront, shiver: negativity as a critique of the “natural caregiver” stereotype in nursing

**DOI:** 10.1590/1980-220X-REEUSP-2023-0129en

**Published:** 2023-10-30

**Authors:** Maria Raquel Gomes Maia Pires, Rebeca Nunes Guedes de Oliveira

**Affiliations:** 1 Universidade de Brasília, Faculdade de Ciências da Saúde, Departamento de Enfermagem, Brasília, DF, Brazil.; 2 Universidade Municipal de São Caetano do Sul, Programa de Pós-graduação em Comunicação, São Caetano do Sul, SP, Brazil.; 3 Universidade Municipal de São Caetano do Sul, Programa de Pós-graduação no Ensino em Saúde, São Caetano do Sul, SP, Brazil.

**Keywords:** Gender Stereotyping, Nursing, Nursing Care, Address, Feminism, Estereotipo de Género, Enfermería, Atención de Enfermería, Discurso, Feminismo, Estereótipo de Gênero, Enfermagem, Cuidado de Enfermagem, Discurso, Feminismo

## Abstract

To discuss, based on Adorno’s philosophy, the negativity of care in confronting the “natural caregiver” discourse in the profession and exercise discursive analysis of this stereotype based on the negative trihedron of care (deny, confront, shiver). Theoretical study that articulates negative dialectic with the biopolitics of caring for the body. Negativity of care, as an immanent criticism that emerges from the dialectic between help and power, aims to shiver at bodily suffering, a residue of nature violated by cultural discursive practices. We applied the methodological framework of care to deny, confront, and shiver in label analysis to highlight non-identity between nursing reality and natural caregiver affirmation. We confronted the injustices made invisible in the prejudice that women are naturally predestined to provide for others’ well-being. We reflected on the contradictions and suffering of women, nurses or not, invisible in the vaunted loving care. We proposed shiver as a metaphor for deny, a critical negativity that opens to the strange coerced and mutilated in the human body.

## RESUMO

Discutir, a partir da filosofia de Adorno, a negatividade do cuidado no enfrentamento do discurso da “cuidadora natural” na profissão; exercitar a análise discursiva desse estereótipo a partir do triedro negativo do cuidar (descuidar, confrontar, arrepiar). Estudo teórico que articula a dialética negativa com a biopolítica do cuidar no corpo. A negatividade do cuidado, como crítica imanente que emerge da dialética entre ajuda e poder, visa ao arrepio diante do sofrimento do corpóreo, resíduo da natureza violentada por práticas discursivas culturais. Aplicamos o referencial metodológico do cuidar para descuidar, confrontar, arrepiar na análise do rótulo para aflorar a não identidade entre a realidade da enfermagem e a afirmação da cuidadora natural. Confrontamos as injustiças invisibilizadas no preconceito de que as mulheres seriam naturalmente predestinadas a prover o bem-estar dos outros. Refletimos sobre as contradições e os sofrimentos de mulheres, enfermeiras ou não, invisibilizados na alardeada amorosidade do cuidado. Propomos o arrepio como metáfora ao descuidado, uma negatividade crítica que se abre ao estranho coagido e mutilado no corpo humano.

## RESUMEN

Discutir, a partir de la filosofía de Adorno, la negatividad del cuidado frente al discurso del “cuidador natural” en la profesión; Ejercer el análisis discursivo de este estereotipo a partir del triángulo negativo del cuidado (descuido, confrontación, escalofrío). Estudio teórico que articula la dialéctica negativa con la biopolítica del cuidado del cuerpo. La negatividad del cuidado, como crítica inmanente que emerge de la dialéctica entre ayuda y poder, pretende hacer que la gente se estremezca ante el sufrimiento de lo corpóreo, un residuo de la naturaleza violado por prácticas discursivas culturales. Aplicamos el marco metodológico del cuidado para descuidar, confrontar y estremecer en el análisis de la etiqueta para resaltar la no identidad entre la realidad de la enfermería y la afirmación del cuidador natural. Nos enfrentamos a las injusticias que se vuelven invisibles debido al prejuicio de que las mujeres están naturalmente predestinadas a velar por el bienestar de los demás. Reflexionamos sobre las contradicciones y el sufrimiento de las mujeres, enfermeras o no, invisibles en el cacareado cuidado amoroso. Proponemos el escalofrío como metáfora del descuido, una negatividad crítica que se abre a lo extraño coaccionado y mutilado en el cuerpo humano.

## INTRODUCTION

At first glance, it seems contradictory to reflect on deny in nursing, a profession that values care as a central part of its knowledge and work. Perhaps because, in general, we readily associate care activities (and, by extension, those who carry them out) as essentially “good”, regardless of the discursive practices and help-power plots in which such care relationships are inserted. Discursive practices correspond to the set of meaning-producing statements that regulate human bodies and actions as well as being regulated by them. As we will see, help-power tension concerns the political dimension of care, present in social relations and vital processes. Thus, it would be worth questioning how the immediate connection between care and kindness was so firmly incorporated into nursing’s sayings, configuring gender oppression^(1–4)^.

On the other hand, if care is always “good”, would we only have to consent to moral dictates, even those that violate us? How do we come to obey rules that confine us to the obligation to provide for others’ well-being (whether older adults, children, sick people, domestic chores or men’s pleasure), based on a presumed determination of feminine “nature”? Part of these benevolences about care – present in discourses that modulate nurses’ and staff’s practice – are moral normalizations about care, said to be “inherent” to women, reverberating in symbolic, material and psychological violence. Among the stereotypes inseparably marked by issues of gender, class, race and generation in nursing, we highlighted the “natural caregiver” stereotype, the object of reflexive disobedience in this study. For that, the quotation marks in the mentioned term consist of a linguistic strategy that intends to highlight the artificial, produced, questionable and changeable character of the determinism to be faced.

The preconceived idea of a “natural caregiver” unfairly prescribes a destiny for women limited to tasks of caring, in view of a supposed and immutable biological determination. This equivocal binary conception of gender, despite the fact that they are social constructions subject to discursive criticism, conditions our bodies to poorly paid and precarious care work, mostly carried out by women, whether nurses or not. This type of work, understood as reproductive or care work by feminist epistemology, maintains the conditions for maintaining human life, including the provision of basic needs and domestic space. Nursing, historically intertwined with the characteristics of this work that reproduces the productive force, also faces perverse working conditions. The profile of nursing in Brazil is wage in jobs (55% in the public sector and 70% in the private sector pay less than 2 minimum wages), precarious employment relationships, multiple jobs, difficulties in obtaining placement in the job market (70%), insecurity, and various forms of violence^(1–7)^.

Currently, women accounted for around 80% of the nursing workforce^(7)^. Historically feminine, for a long time, nursing restricted the participation of men and spread moral judgments on the presence of sex workers, black women or women with low education in care activities. As a result, in addition to maintaining low wages, the category weakened its political organization under an alleged moral superiority of nurses in relation to technicians - supported by a positivist scientism that blunts critical thinking. In other words, when we incorporate capitalism’s patriarchal sexisms into the nursing team’s social and sexual division, we mutilate the category’s political quality and power to pressure for working conditions, despite being a quantitative majority. This look at the insertion of women in the job market reveals the production of stereotypes that mark nursing, which oscillate between the figure of an angel (sacred) and a prostitute (profane), reinforcing “natural caregivers’” many faces in nursing^(1–6)^.

Literature reviews identify the reverberations of gender stereotypes in society’s judgments about nursing, including: for female nurses: technical incompetence; poor academic and professional level; incipient autonomy; and body sexualization; for male nurses: questioning masculinity; balance of the same violence against women^(8)^. We asked, however, whether these stereotypes are not also fed back by impregnations of a “natural caregiver” in nursing discourses. In this regard, contradictions in female nurses’ voices and gestures are emblematic. The reasons for professional choice, for intsnace, for women continue to be marked by sexism, rigid moral and religious values, such as altruism, family incentives or illusory idealizations of “being a nurse”. For those who identify as men, the motivation for the profession is marked by values such as leadership and scientificism, demarcating sexism in gender roles^(9)^.

In the academic environment, a curious analysis of a “care fee” paid by fellow nurses in the workplace – namely, a certain emotional toll arising from the moral transposition of maternal and family relationships to university institutions, permeated with similar veiled aggression – exemplify how little “kind” the incorporation of a “natural caregiver” can be among us^(10)^. Furthermore, nurse researchers’ speeches often reissue, without any criticism, sexist ideologies that conceptualize nursing as “a human activity linked to women”, in which a supposed “maternal instinct” would provide “motivation and necessary impulse to care”^(11)^. Likewise, we saw the “natural caregiver” re-edition in training, even in initiatives that aim to question stereotypes. A study in which sexualization of nurses, male leadership, emotional fragility and care as a feminine attribute were identified in the “views of society” by students showed that reflections about our counterpart in maintaining these gender stereotypes went beyond reflections^(12)^.

In contrast, there are productions that criticize “natural caregivers’” discursive problems in nursing, epistemic resistance that needs to gain visibility and strength, in a clash capable of reverberating over practices. With a suggestive title of “fallen angels and forgotten heroes”, a survey investigated heroic idealizations in nursing professionals’ perceptions during the COVID-19 pandemic, noting how distant these images are from nursing daily care according to interviewees. On the same topic, similar results were discursively analyzed in a Brazilian study^(13,14)^. In the recent health crisis caused by the pandemic, studies problematize the media’s appealing sentimental tributes to nurses and staff, which did not have any impact on achieving the minimum wage for the category – nor on reducing social inequalities in nursing–, which continues to be immersed in pauperization that inhibit collective action^(15–17)^. Despite resistance, we found gaps regarding the uncritical impregnation of stereotypes of race, class, gender and generation in nursing’s scientific discourse. In other words, there is a clear disconnect between what nursing claims to be in relation to its daily life and the result of self-illusory idealizations little reflected in studies in the area^(10,15,16)^.

In this context, it is urgent to neglect “natural caregivers” in nursing, as such totalitarian identifications reiterate nursing’s political, symbolic and material weaknesses. The proposed deny begins with the ability to say “no” to symbols that restrict the female condition to a generalization of how we should be, do or behave. As an example of neglect that we need to expand, here is a campaign promoted by professional associations in the state of Alagoas during the pandemic, whose motto was “Neither angels nor heroes. We are professionals, we are NURSING”^(14)^.

In theoretical terms, negativity constitutes the dialectical moment that opposes the positive absolutism of concepts, i.e., it denies that they can fit into things like gears, whose most glaring contradiction are stereotypes; in short, it seeks non-identity between words and things, given the insufficiency of language to say what things are in themselves^(18)^. Negative dialectics fights against consciousness reification (objectification), remaining critical and self-critical of affirmative syntheses that prevent changes. Alongside this reflection, denial of gender stereotypes in nursing consists of a deny of judgments that dictate behaviors to women as “natural” – synonymous with fixed, immutable, unconditioned –, resulting in intersectional inequalities^(18,19)^.

We conceived care as correlations of integrative and disruptive forces of complex social relationships, whose practices are forged and modulate discourses. In the politics of care, there is tension in the links between help and power – the latter seen as a strategic situation of forces and counterforces centered on the biopolitics of bodies. The conception of help here is based on Marcel Mauss’ gift trilogy, seen from the perspective of power, in which the moral obligation to give, receive and reciprocate strengthens the processes of cultural formation of different ethnicities. Thus, every gesture of care occurs amidst tense and asymmetrical correlations of forces, since whoever offers help, even in order to be able to do so, ratifies a hierarchical position in relation to whoever receives or needs help. Seen as a field of forces, a power is supported by counter powers that can subvert it, depending on the politics in dispute. In other words, the resistance of one power by another power, also called negativity or criticism, begins a rupture with an unfair asymmetrical situation, with which we do not agree, such as that of “natural caregiver”^(1,4,18,19)^.

The present study is a theoretical-philosophical reflection on negativity of care – expressed in care to deny, confront and shiver – in the face of “natural caregiver” repercussions in nursing discourses. The care trihedron is based on the methodological framework of in-depth hermeneutics for analysis of ideologies, adapted to discursive practices of care^(1,20)^. In this negative version of the trihedron, we included the term shiver on the third face based on “shiver” in Adorno, based on the moral interpretations of negative dialectics. Meanwhile, since “shiver” can happen when faced with aesthetic or moral objects, it is not a question of adopting Adorno’s aesthetic theory in the politics of care. Without entering into a debate that goes beyond the purposes of this text, we conceived the term solely to speak of the sensation of similar bodily shivering in the face of contradictory perceptions of speech when confronted with the characteristics of things in themselves^(18-22)^. In other words, we defend negativity as a discursive criticism that makes us shiver in the face of reflections about the conventional oppressions of “natural caregiver” in ourselves and in others – keeping the outcomes open at the dialectic’s negative pole.

From the above, the guiding question of this study is: in the dialectical relationship between help and power inherent to the politics of care, in what way can negativity criticize the “natural caregiver” stereotype in nursing discourses? We argued that, in the negative moment of care, the dialectical relationship between help and power coincides with the shivering in the face of bodily suffering, a residue of nature violated by discursive practices, constituting a critique of identity statements between “natural caregiver” and nursing. The objectives are to discuss, based on Adorno’s dialectics, the negativity of care in confronting “natural caregivers’” discourses in nursing as well as exercising analysis of discursive practices of this stereotype based on the methodological framework of the negative trihedron of care (deny, confront, shiver). In the first topic, *Caring to neglect: negativity as discursive criticism*, we articulated Foucault’s biopolitics with Adorno’s negative dialectics and the conception of politics of care, within the scope of the discursive critique of suffering. In the second topic, *Negative trihedron of care: deny, confront and shivering of “natural caregiver” in nursing*, we applied the methodological framework of the negative trihedron of care in the discursive criticism of this stereotype in nursing.

### Caring to Deny: Negativity as Discursive Criticism

But how do the biopolitics of care (administration of bodies, calculated management of life, powers that organize the body) and negativity as a discursive critique of suffering articulate? Why can negative dialectics confront discourses that shape practices? What criticism are we talking about? What does suffering consist of as a residue of nature violated by discursive practices, within the scope of culture? Can the negative moment of dialectics in care resist and combat discursive violence, such as that of the stereotype of a natural caregiver? In short, where do biopolitics and negative dialectics of politicity of care meet? Answer is: in the body which is perishable, vulnerable, changeable, object and agent of care. A discursively shaped body, disciplined and turned over – also rebellious, product and producer of power devices^(18,19)^. We will explain these questions, not necessarily in that order.

The dynamics of help and power of care operate in the body, supported by scientific biomedical and nursing discourses that prescribe it. Care as biopower, confronted by other powers, penetrates and controls the use of pleasures in the body. This care exercises power and takes pleasure in turning over, revealing, undressing, manipulating, objectifying and telling the truth about the body, which rebels. Biopower, mediated by care, disposes, controls and produces disciplined bodies under diffuse, indocile somatic protests; it regulates sexuality under the subversions of pleasures. Care as biopower makes the body docile; conceives it as a machine; classifies it as normal or pathological; produces speeches and prohibitions; has a desire to know amid resistance from diffuse speeches and drives. Biopower is a device of unstable, strategic, multiple and disruptive power over life; it also intervenes, controls, produces and cares for populations, hence the implication of nursing in knowledge production about caring for the body^(18,19,23)^.

By conceiving care as a dialectical tension between help and power, understood as a structural component of human relationships, we abandoned the moral rigidities surrounding care as always “good”, altruistic, benevolent and similar adjectives. This is justified because both Foucault’s biopower and help based on Mauss’s gift trilogy (obligation to give, to receive, to reciprocate) inseparably make up social relations permeated by ambivalence and relativization^(4)^. As a result, care follows the flexibility of human bonds in their political character, under different aspects. Within the scope of discursive practices, care is sometimes revealed as work and profession, sometimes as a moral obligation, sometimes as charitable help without material reciprocity purposes, in a cyclical and complex interdependence permeated by the effects of the power that constitutes it. In other words, however it manifests itself more visibly, care constitutes the power relations structured by discourses that shape it, as much as it feeds back on these meaning-generating statements^(1-5,19)^. It turns out that such devices leave indelible, hidden, unconscious and somatized marks on the body, sometimes under complex syndromes of diffuse illnesses and suffering. Likewise, the body suffers the setbacks of violence caused by the discursive biopolitics of gender stereotypes, which we want to confront and shiver due to negativity of care. To understand this point, we will talk at least about the structuring of language based on the process of domination of nature, a central theme of the Dialectic of Enlightenment^(24)^, by Adorno and Horkheimer.

According to Critical Theory philosophers, faced with the primordial fear of mythical and threatening forces of nature, human beings strive to tame them in order to survive, mainly through the use of language to designate things. In other words, through language they produce declarative statements to explain the forces of nature, with the aim of containing them. Thus, they want to correspond what is said to what is said, reporting objectively to phenomena with statements about them. But how does the designation of objects reduce primordial fear? In the process of identifying the things that frighten them through language, human beings make known what they were previously unaware of (or at least generate meaning about them), being able to act on their mythical fear^(24)^.

An important detail is that in modernity this designative subject is hegemonically male, heterosexual, white, Eurocentric, in which women appear as the “other” of positive reason. In other words, what men have always wanted to dominate, taken by fear (impulses, nature, emotions), historically outlines the sexist representation of the female body, producing oppressive discourses, hence our implication in neglecting structural gender violence^(23-25)^. Under the pretension of reason to constrain natural forces, contradictorily, in the desire to submit it, human beings fell dominated by it, externally and internally, sinking into a kind of “barbarism”. In terms of the Dialectic of Enlightenment, instead of enlightening the world to overcome obscurantism through emancipation of reason, the universal male subject fell into the darkness that he believes he controls, resulting in the Holocaust.

The episode of Ulysses and the Sirens, in Homer, was used as an allegory of this domain of nature mediated by language in Adorno and Horkheimer. The excerpt is well known: Ulysses, Greek hero of the Odyssey, on the long voyage home, faces all kinds of dangers, dodging them shrewdly. In one of the adventures, he was warned by Circe, a sorceress, about the sirens’ song and how to survive it, since men dive into the sea and succumb, enchanted by desire. The prophecy fulfilled, upon encountering the first melodic breaths, Ulysses orders the crew to put wax in their ears so that they cannot hear them and row without stopping. He ties himself to the mast, but leaves his ears open to hear the beautiful singing. Everyone escapes. It seems that the undertaking was successful and guarantor of human life, since it was guided by a reason that is not distracted by arts, myths, women or enchantments.

But at what cost do we give up poetic sensitivity, desires, dreams and aesthetic charms – as present in our internal nature as aggressiveness – to preserve ourselves, under the auspices of scientific discourses’ instrumental language (pragmatic, rational, objective)? What suffering do we archetypically carry in our bodies, a remnant of the violence of this civilizing process to docilize the human animal? It is not the aim here to delve into the excerpt’s complexity and multiple interpretations, which include subject formation, consciousness reification, desire for modern self-preservation and its consequences, extirpation of poetic sensitivity from language, imprisonment of art, usurpation of reflective capacity, among others^(24,26)^. We will highlight the process of instrumentalization of language and physical violence that interests us.

In one of the readings, the fact that Ulysses orders the occlusion of the crew’s ears with wax – so that they do not hear the sirens’ song – is related to anesthesia of senses in language, in the search for scientific knowledge objectivity and subject separation (man, nominator of things) in relation to the object (subjugated, named nature). By curbing sensitivity, science better controls repeatable phenomena, under the pretense of neutrality. With disenchantment, scientific language becomes aseptic, without smell, voice, image, song, much less magic; its usefulness is calculable, comparable, standardizable – just like nature and the beings it measures. Deprived of the affectation of the sensible, science impoverishes the myth, making it a manipulable dogma. We explain: it dissects the mythical, poetic, imagery, allegorical and aesthetic narrative of language, reducing it to eliminable magic.

We also see in the excerpt the process of domination of human beings’ external and internal nature through social division of labor, with consciousness reification (automation) and utilitarianism (they row as if there were no tomorrow). Ulysses’ initiative to enjoy the sirens’ song without destroying himself – obtaining what he wants from nature, although as mutilated as the others – exemplifies a supposed superiority of thinking over doing, for instance. Contradictorily, in the quest to overcome the fear of sensual impulses (sirens with their art, muses; we women, abhorred as carriers of destructive desire over men), instrumental reason succumbs to the same violence that it inflicted against external nature; this is the Dialectic of Enlightenment. In other words, if the intention was to overcome the myth, science itself becomes a myth, only dissected and devoid of poetry^(24)^. It would be worth asking what sirens promise men that are so irresistible that they sacrifice their lives. One of the interpretations focuses on knowledge, as they know everything there is in the world^(26)^. Under the paradox of enlightened reason, in the fear of losing themselves in a nature that also belongs to them, human beings produce science about a nature that they believe they dominate through language, appearing more frightened than before, perhaps even more ill.

Understanding the repercussions of the domination of nature on the body, it goes without saying how much suffering as a residue of the discursive interdiction of what we cannot control (impulses, desire, nature, ineffable anguish) is expressed in the body under complex illnesses. Take, for instance, cancer, panic syndromes and others, psychological disorders and, why not, pandemics caused by viruses expelled from their natural habitat that acquire the capacity to infect humans. It is essential to note that the rationality of science, on which nursing is nourished, is difficult to deal with bodily suffering in any other way than this: classifies symptoms; reduces people to pathologies and compares them; measures, medicalizes, positives and numbs pain, which returns sicker.

We cannot bear pain; we do not hear it in ourselves or in others; much less do we shiver at its violated nature. With this, we reduced care to biomedical procedures instead of experiencing it as a cure. We divided pleasure and pain under infallible formulas of instrumental reason’s positive sciences (coaching, cognitive psychology, others)^(27)^. We survived mutilated under the positivist discourse that ratifies the historical contradiction between care and capitalism, i.e., to accumulate, capital depends on reproductive work that devalues and stigmatizes^(5)^. A provocation would be: would not a profession that prides itself on caring, such as nursing, be at the center of this debate? And what do we know or neglect about it?

Recapitulating, the notion of bodily suffering consists of residue of nature violated by discursive and biopolitical practices embedded in us, shaped and shaping civilization. The traumas of denial of animality, of desires, of corporeal playfulness by culture are unconscious, singular, without language. The more violently we reject aggressiveness (along with sensitivity) – under the so-called moral equivocations of benevolence, compassion, selflessness, love and perfectibility – the more hatred we produce. Resentment, i.e., everything that we do not expiate and feel back in the body, under psychosomatic manifestations, resulted in the exclusion of the other, of nature, of the imponderable and unclassifiable non-identical that scares us, as much as it belongs to us. We only need to look around to realize that we continue to deal with the problems of resentment. We do not need much to see that women as “the other of reason”, also known as the body, emotions, nature and sexuality that divert men from their straight path, continue to be the target of oppression based on gender, race, class and generation conditions^(23,25)^. It is against the totalitarianism of reason and identifying, positive, masculine language, unifying the particular in the universal under labeling discourses that negativity is placed. It will now be addressed.

Negative dialectics “has the task of pursuing the inadequacy between thought and thing; trying it on the thing”^(18)^. It addresses the diverse, the non-identical imprisoned in concepts and prejudices. Procedurally, it thinks about contradiction, places itself for and against it, turns to what cannot be named in order to place “the identical under suspicion”^(18)^. In the dialectical movement, denial presents itself as a questioning of social immediacy, placing it under criticism based on the characteristics of the object itself.

With negation, statements come into contradiction with what they affirm, as “objects do not dissolve into their concepts”^(18)^. Under the Hegelian maxim that thinking is denying, Adorno proposes the impediment of the moment of dialectical synthesis so that difference destabilizes identity in a negative proposition of change. In the negative of thinking, the aim is to save the contradicted, even though through concepts. Contradictorily, when we deny statements about something, what was denied remains contained in the denial. For instance, when we say no to the natural caregiver stereotype in nursing, we affirm this denial, so it also becomes positive. The difference is that negative dialectics is aware of this paradox and works with it, seeking it as a way of approaching the unprecedented.

Therefore, negative dialectics is the “consequent consciousness of non-identity”^(18)^; it seeks object primacy (nature; the body; the woman; the phenomena; the difference) where the naming subject (man, white, heterosexual, Eurocentric; the equal) abounds, without dismissing them. In a return to materiality, to the body, by throwing itself at things, negative dialectics takes on the task of presenting what “this something”^(18)^ of the world is, forcing conceptual antinomies. In the experience of resisting what is imposed, the condition of dialectical thinking makes it necessary to give vent to suffering, because “true are only thoughts that do not understand themselves”^(18)^.

We then arrived at the non-identical of negative dialectics: the pain of nature repressed in the civilizing process, always refractory to the affirmative conceptual discourse of science. This pain is located in a body of precarious lives, organized by controlling biopolitics that, being power, are also disorganized in and by the body^(18,19,23)^. Negativity is the thought that says “no” to what appears immediately given to the senses as immutable, necessarily determined in one way, and not another. This is the privileged movement of criticism, without which changes cannot be generated.

Certainly, this is not about any criticism, or any that comes from outside (in this case, prescribing that women are natural caregivers in view of biology by external dogmas, disregarding the particularities of each one of us), but about immanent criticism, i.e., which is inherent to the object. In other words, immanent criticism rejects any criteria external to what it intends to describe; it searches for potentials in the object itself, through them it carries out self-reflection, incites the difference between the thing and the concept. It explains the distance between what a thing can be in relation to what is said about it, or what it can be. The negativity of immanent criticism confronts discourses about phenomena in relation to things in themselves, but seeks the contradictions of non-identical defined as equal or similar without, however, seeking a new identical, since it avoids the positive conceptual synthesis identified^(18,21,22)^.

By way of synthesis, we said that biopolitics intersects with negative dialectics in the body as an object and agent of care conceived in the subversive tension between help and power that shape discursive practices. We feel in our bodies the unspeakable marks of our violated and suffering vulnerability, arising from the domination of external and internal nature in the civilizing process, mediated by the structuring of language in human actions. In the Dialectic of Enlightenment, we realized that human beings have incorporated the very primordial fear that they wanted to destroy externally, producing more violence and a barbarized version of ourselves.

With the instrumentalization of human reason, expressed in scientific language linked to capitalism, the bodily suffering of nature violated within us manifests itself under complex illness syndromes that challenge biomedical and nursing knowledge. Therefore, in general, our care is reduced to procedures; our knowledge accumulates evidence and avoids contradictions; our practice incorporates and reissues discourses that violate us under the epithet of “natural caregiver”. We saw that negative dialectics seeks precisely what was relegated in the process of positivization of declarative language, namely: the non-identical, the violated nature in the body. Deny as an immanent critical negativity of discourses then contrasts the concept with the thing, the object with what is said about it, causing ruptures and disintegrations of a supposedly immutable whole.

We will see, in the next topic, how the negative trihedron of care – expressed by care to deny, confront and shiver – carries out the discursive criticism of gender stereotypes in nursing, taking “natural caregivers” as an instance.

### Negative Trihedron of Care: Deny, Confront and Shiver of a “Natural Caregiver” in Nursing

Immanent criticism carries out the denial of ideology, seen as a “socially necessary illusion”^(18)^ or as a discourse that legitimizes power in society. In Adorno, ideological discourse concerns the inseparability between the particular (difference) and the universal (equal), a product of enlightened reason. In other words, ideology takes human singularities for generalities that socially maintain hegemonic powers, discriminating people according to discourses conveniently produced and established by power relations. From this perspective, criticizing gender stereotypes also means criticizing sexist, male chauvinist and racist ideologies that reproduce the contradictions between capitalism and care, which devalue the work carried out by women. Therefore, the natural caregiver stereotype in nursing feeds into other discriminatory labels based on binary conceptions of gender. If we look carefully, nursing historicity is confused with this miscellany of prejudices that inform the gender category, given the social and sexual division of work present in the team, unequally marked by conditions of race, education, social class and generation between nurses and techniques^(4–7,13–17)^.

Another highlight regarding immanent criticism concerns its insertion in Adorno’s moral philosophy, especially regarding the denial of bodily suffering. In other words, “the bodily moment announces to knowledge that suffering must not be, that it must change. Pain says: perish”^(18)^. It is understood that this denial of suffering (the non-identical nature violated in the body) does not mean its anesthesia, as in the biomedical model discussed above. Nor could it, since pain as negativity constitutes “the engine of dialectical thought”^(18)^. Nor does it refer to a concern restricted to physical pain, regardless of psychological, moral and other pain. Unlike this, what is proposed is the critique of suffering that is somatic, material, but also meaningless, i.e., inhuman.

The bodily vibration of meaningless pain becomes a relevant moral criterion to identify the evil to be combatted. For Adorno, since we cannot know what good is without falling into dogmatic discourses, suffering informs at least what we do not want, what cannot be conceived for human life, what connects us to others through primal suffering, experienced singularly^(18,21,22)^. Thus, society “[…] would have its *telos* in the denial of physical suffering of even the last of its members and in the forms of reflection intrinsic to this suffering”^(18)^.

In this context, at the intersection of negative dialectics with the body biopolitics, deny initiates the negativity of bodily suffering or enables the “forms of reflection”^(18)^ somatically mobilized by pain to gain magnitude in dialectical thinking. Deny as an immanent discursive criticism seeks the semantic contradictions of discourses through denial of prefixed statements, causing thing characteristic inadequacy with the false label received. Within the scope of nursing practices, we do not need much effort to see that nurses’ and technicians’ daily lives suffer from the oppressive reverses of the supposed benevolence of care, due to work overload, low wages (especially in care, where the “natural caregiver’s” imagination prevails), difficulty of employment or intersectional discrimination previously discussed^(7–17)^.

In the same way, the discourse of “nature” present in the natural caregiver stereotype should be ignored. Due to the sexist bias that supports it, women would be destined to care for their biological “nature”. However, what nature is it when this alleged immutability is attributed? The deterministic, evolutionary nature, restricted to anatomopathological characteristics, divided between the virile male and the fragile female, only encourages sexist desires as much as the mechanistic and Darwinist discourses, long refuted by science. The nature that we do not know, as much as we wish to dominate through language, denies this version: it is mysterious, chaotic and organic at the same time, uncertain, unpredictable, ambiguous, beautiful and fierce, whose exuberance of forces terrifies, seduces and reminds us of our defenseless, fragile and powerless origin^(18–26)^.

But if science and discursive analysis itself deny this version of a rigid and sexist nature, where would the discourse of women come from as being inferior to men, fragile, defenseless, emotional, maternal and caring in essence? In this regard, it is not difficult to remember the influence of philosophical and theological theories of natural law, or justanaturalists, according to which nature and creatures as divine creations follow a greater law that orders them hierarchically, and it is up to human beings to obey it and never modify it. This same vision justified the bloody process of Brazilian colonization by white European men as well as slavery of black people, sexual exploitation of black women and evangelization of indigenous people, as it was doubted that they would have souls^(28)^. The residue of all nature violated by discursive ideologies is incorporated into us without language, whose memory escapes consciousness. We see yet another contradiction inherent in “natural caregivers’” discourse, since nature itself can be anything but immutable as well as any “feminine essence” conveniently discussed to appropriate our bodies^(2–6,23–26,28)^.

Once we have semantically neglected the “socially necessary illusions” for maintaining natural care, we compared the immanent characteristics of nature with what is said about it. We contrasted the perverse meanings that this stereotype produces about women’s bodies and voices in the work of care, so attached to the knowledge and practice of nursing. We also analyzed the violence that the belief in “natural caregiver” produces, by ratifying the sexist ideology of the docile, obedient, affectionate, selfless, renounced, modest, maternal, devoted woman dedicated to meeting men’s needs. In nursing, this view of women is called “angels and heroes”, practitioners of “care for love”. In this photolith, here is the negative point: if we are angels, we do not even have a body or needs, which performatively contradicts the sexist stereotype that women represent the voluptuousness of bodily desires. So, we, nurses and technicians, do not need a basic wage, as flowers and formal smiles once a year as well as “clapping from windows” remunerate us. The phrase’s irony is proportional to the remnants of suffering caused by thoughtless obedience to ideologies that dehumanize us.

After denying and comparing “natural caregivers’” discourse with nursing reality, we arrived at the third, most uncertain, negative and ambiguous face of the trihedron, the shiver. Shiver is an unpredictable, sudden, uncontrollable and non-specific physical sensation that causes concomitant feelings of pleasure and discomfort in the body. By proposing shiver to the analysis of discursive practices through the care trihedron, we intended to associate this unusual physical sensation with the foreshadowing of the somatic non-identical, i.e., the imprisoned nature that calls for indeterminate manifestation.

Just as the reasons that cause shiver in the body are diverse, individual and non-standardizable, in the same way, in negative dialectics, shiver does not mean synthesis or emancipation of care. On the contrary, it denies precisely this coupling; before that, shiver refers to “[…] naked and raw physical fear and the feeling of solidarity with the torturable bodies”^(15)^. In other words, like shivering, shiver physically signals that others’ suffering mirrors our own wounded humanity, even if suddenly. The human solidarity of caring about others makes up the characteristics of moral behavior that had been “denied through aspiration to uncompromising rationalization”^(18)^; it should be read: restrained by affirmative instrumental reason.

Thus, detailing this point, it is not about classic compassion, the one that feeds moralistic discourses of kindness and love that cover up the injustices of the care work provided by women^(2-6)^. Nothing is further from negative dialectics than universalization of norms with a tendency to dogma, whatever they may be, including the unreflective inconsistencies of positivist science. Instead, what would drive this feeling of inclusion of the other, of mutilated and voiceless stranger, would be a common remnant of human terror and pleasure in the face of natural forces, of non-identical enclosed in bodies in the process of domination of nature. In other words, body tremor reminds us of the similarity and desire for the exuberant and fearful external and internal nature that has been violated (remember the sirens in the myth of Ulysses). Certainly, human impotence and fear in the face of the magnitude of phenomena that we cannot control, mixed ambiguously in feelings of pleasure and pain, although numbed by medication, would be less than the fear of definitively losing the tremors that vivify us, hence the bet on shiver.

Shiver is a unique sensation, but this ability is humanly shared. Furthermore, what makes us morally human – in other words, singularly prone to caring for others, to the desire to welcome the stranger who lives within us – involves the ability to shiver in the face of suffering, inhumanity, and precariousness of life^(29,30)^. In the momentary shock of shiver, depending on the excitement provoked, the negativity and disobedience of unworthy norms increase or not.

Obviously, human motivations that provoke impulsive changes in moral action are unpredictable, especially with regard to the biopolitical possibilities of care that confronts ideological discourses. Nor will we prescribe moral conduct based on shiver, since we would fall into the positivity of the reified thought that we contest. We know that a shiver can be pleasant or uncomfortable, strong or weak, more or less intense – but this physical discomfort rarely goes unnoticed. Likewise, in the negative trihedron of care proposed here, the shiver sought is what causes us to open up to the stranger, in such a way that it is difficult for us to remain indifferent to the sensation provoked. Whether or not we provoke shiver when confronting the suffering caused in ourselves and other women by natural caregivers’ fallacies, it is a risky, negative move, subject to multiple interpretations.

### Final Considerations

In the encounter between biopolitics and the negative dialectic of care in the body, we argued for a negative trihedron of care to the natural caregiver stereotype discursive critique in nursing and its repercussions on practices harassed by prejudiced discourses, sometimes ratified in the voices of nursing. From caring to deny, confronting, shiver, we carried out discursive analysis to surface non-identity between the concreteness of nursing practice and what the label of “natural caregiver” states. In deny, we carried out the immanent criticism of both nursing reality and the notion of nature vaunted in woman care. With this, we stirred up the disagreement between nurses’ and staff’s inhospitable daily lives in relation to stereotype fallacy. In other words, with deny, we reflected on women’s contradictions and bodily suffering, nurses or not, invisible in the vaunted loving care. Having exposed the inconsistencies that harm more than care in the natural caregiver stereotype, we provocatively contrast these versions, in order to combat the discursive hegemony that women are predestined to provide for others’ well-being. The shiver proposed here, felt physically or not, consists of an associative metaphor for discursive negativity – a critical deny that seeks, without coercing, bodily suffering, a residue of nature violated by discursive practices. If we have physically awakened any feeling of discomfort – whether in those who doubt or disagree with this text – it will be our turn to shiver.
